# Beta-adrenergic signaling, a novel target for cancer therapy?

**DOI:** 10.18632/oncotarget.182

**Published:** 2010-11-11

**Authors:** Hildegard M. Schuller

**Affiliations:** Experimental Oncology Laboratory, Department of Pathobiology, College of Veterinary Medicine, University of Tennessee, Knoxville, USA

In the current issue of *Oncotarget*, a 10-year follow-up study by Powe et al. reports that women receiving beta-blocker therapy for hypertension showed a significant reduction in breast cancer metastasis, recurrence and mortality. These findings provide first clinical evidence in support of earlier in vitro findings by the same research group reporting significant norepinephrine-induced stimulation of cell migration that was inhibited by the beta-blocker propranolol or by the γ-aminobutyric acid (GABA)-mediated reduction in cAMP-dependent signaling in cancer of the colon, prostate and breast [1-4]. Additional breast cancer-stimulating mechanisms that may have been inhibited by beta-blocker therapy include beta-adrenergic receptor (β-AR)-mediated proliferation of cancer cells and the β-AR-mediated activation of the arachidonic acid (AA) cascade as well as β-AR-mediated modulation of G-protein inwardly rectifying K+-channels observed in vitro studies by another laboratory [5,6]. Collectively, these findings identify signal transduction pathways downstream of β-ARs as important positive regulators of progression and metastasis in these cancers and represent a promising new target for the therapy and prevention of such malignancies.

Beta-ARs are a family of G-protein coupled receptors that initiate multiple signaling cascades, including the adenylyl cyclase/cAMP/PKA/CREB pathway which transactivates the epidermal growth factor receptor (EGFR) pathway, the Src/STAT pathway as well as the arachidonic acid (AA) cascade [7-11]. The catecholamine stress neurotransmitters noradrenaline and adrenaline (synonyms: norepinephrine and epinephrine) are the physiological agonists for β-ARs [12]. These neurotransmitters are not only released from the adrenal medulla as a response to psychological and physical stress but also regulate cell and organ responses to the sympathetic branch of the autonomic nervous system [13,14]. In turn, the synthesis and release of noradrenaline and adrenaline in the adrenal medulla and sympathetic nerves are regulated by nicotinic acetylcholine receptors (nAChRs)[10]. On the other hand, the neurotransmitter GABA serves as the physiological inhibitor of β-AR signaling by blocking the activation of adenylyl cyclase via the inhibitory G-protein (Gα_i_)-coupled GABA_B_ receptor [15].

First evidence for a regulatory role of β-ARs in cancer cells was provided in 1989 by an in vitro study that showed a significant increase in the proliferation of human lung adenocarcinoma cells in response to the β-AR agonist isoproterenol with the general β-blocker propranolol inhibiting this response [16]. Later investigations revealed similar epinephrine-induced effects in these cells that were blocked by propranolol and were depended on β-AR mediated increase in intracellular cAMP [17]. Discoveries that the nicotine-derived and highly carcinogenic nitrosamine (4)-methylnitrosamino-1-(3-pyridyl)-1-butanone (NNK) is a high affinity agonist for β1-and β2-ARs [18] as well as for nAChRs [19] provided a first etiological link between the activation of nAChR-regulated β-AR signaling and the development of smoking-associated cancers. It has thus been shown that NNK stimulates the proliferation of human lung adenocarcinoma cells and their normal cells of origin, small airway epithelial cells, via a cAMP-dependent signaling cascade that includes activation of the transcription factor CREB as well as the PKA-dependent transactivation of the EGFR pathway and the β-AR-mediated release of AA [9,18,20]. This signaling cascade was not only stimulated by direct binding of NNK to β-ARs [18] but additionally by the α7nAChR-mediated production of noradrenaline and adrenaline in small airway epithelial cells and lung adenocarcinoma cells [21]. These in vitro findings were further corroborated by reports that epinephrine promoted while propranolol inhibited the development of NNK-induced lung adenocarcinomas in hamsters [22]. In addition, chronic nicotine-induced progression of xenografts from a human lung adenocarcinoma cell line was associated with increased systemic levels of stress neurotransmitters and upregulation of nAChRs, cAMP, p-CREB and p-ERK in the tumor cells while treatment of the mice with GABA inhibited all of these responses. In analogy to these findings, it was also shown that cell lines from pancreatic ductal adenocarcinomas were stimulated in their growth via NNK or isoproterenol-induced β-AR signaling [23] and that the beta-blocker propranol inhibited the development of NNK-induced pancreatic cancer in hamsters [24] while inducing apoptosis in pancreatic cancer cells in vitro [25]. Moreover, nicotine induced significant progression of pancreatic cancer xenograft growth that was associated with a significant systemic increase in stress neurotransmitters and upregulation of cAMP p-CREB and p-ERK in xenograft tissues. In turn, GABA blocked xenograft growth and the associated upregulation of cAMP-dependent signaling in response to nicotine [26]. Cell lines from human gastric and colon cancers also produced noradrenaline in response to nicotine treatment and the resulting increase in cell proliferation and angiogenesis was inhibited by propranolol [27-29]. It hence appears that the most common human cancers are not only stimulated by stress neurotransmitters in the systemic circulation but additionally produce their own noradrenaline and adrenaline.

Emerging research additionally suggests an important stimulating role of psychological stress in the progression, angiogenesis and metastasis of numerous cancers. It has thus been shown that social stress in mice increased the metastasis of breast cancer xenografts [30] and that stress induced by passive restraint as well as treatment with epinephrine had similar effects on ovarian cancer [31] while social isolation stress of mice significantly increased angiogenesis and metastasis of colon cancer xenografts [32,33] . Interestingly, treatment with propranolol significantly reduced these effects. Moreover, recent studies with lung adenocarcinoma xenografts have shown a significant stimulation of tumor progression in mice exposed to social stress, an effect associated with upregulation of nAChR subunits alpha7 and alpha4 and accompanied by systemic increase in stress neurotransmitters, reduction of GABA and activation of the cAMP/P-CREB/ERK pathway in tumor tissues [34]. Mechanisms such as this may contribute to the ethnic and racial differences in the smoking-related risk of lung cancer [35] while additionally providing a mechanistic explanation for reports that psychological stress is a significant predictor of lung cancer mortality [36] and that lung cancer patients with a high rate of psychological distress at the time of diagnosis also have a history of pre-existing psychological stress [37]. In addition, the anxiety of patients diagnosed with cancer represents an additional form of stress that may counteract the efficacy of cancer therapy. It has thus been shown in mouse models of post-operative metastasis that propranolol and a COX-2 inhibitor given before and after surgical removal of primary melanoma cell or Lewis lung carcinoma cell foot pad implants had significantly fewer post-operative metastases than controls [38].

In summary, beta-blockers that have been safely used as cardio-vascular therapeutics for decades, and GABA that has been a safe nutritional supplement for many years, significantly reduce the proliferation, progression, angiogenesis and metastasis of the most common human malignancies, including adenocarcinoma of the breast, lung, pancreas, prostate, colon and stomach as well as ovarian cancer [39]. These agents should therefore be rapidly moved into clinical applications to increase the efficacy of currently available cancer therapeutics and cancer surgery. In addition, nutritional supplementation with GABA should be explored for the prevention of cancer in individuals at risk (e.g. current and former smokers).
